# Chimeric antigen receptors for adoptive T cell therapy in acute myeloid leukemia

**DOI:** 10.1186/s13045-017-0519-7

**Published:** 2017-08-29

**Authors:** Mingxue Fan, Minghao Li, Lipeng Gao, Sicong Geng, Jing Wang, Yiting Wang, Zhiqiang Yan, Lei Yu

**Affiliations:** 1 Institute of Biomedical Engineering and Technology, Shanghai Engineering Research Center of Molecular Therapeutics and New Drug Development, School of Chemistry and Molecular Engineering, East China Normal University, NO. 3663 Zhongshan Road, Shanghai, 200062 People’s Republic of China; 2 China Novartis Institutes for Biomedical Research Co., Ltd., GDD/TRD/Chemical and Pharmaceutical Profiling, 5F, Building 3, Novartis Campus 4218 Jinke Rd, Zhangjiang Hi-Tech Park Pudong District, Shanghai, 201203 China

**Keywords:** Chimeric antigen receptors, Acute myeloid leukemia, Immunotherapy

## Abstract

Currently, conventional therapies for acute myeloid leukemia (AML) have high failure and relapse rates. Thus, developing new strategies is crucial for improving the treatment of AML. With the clinical success of anti-CD19 chimeric antigen receptor (CAR) T cell therapies against B-lineage malignancies, many studies have attempted to translate the success of CAR T cell therapy to other malignancies, including AML. This review summarizes the current advances in CAR T cell therapy against AML, including preclinical studies and clinical trials, and discusses the potential AML-associated surface markers that could be used for further CAR technology. Finally, we describe strategies that might address the current issues of employing CAR T cell therapy in AML.

## Background

Acute myeloid leukemia (AML) is a cancer of the myeloid line of blood cells that is characterized by the clonal expansion of abnormal myeloid progenitors in the bone marrow and peripheral blood, which interferes with the normal production of blood cells. AML is a rare disease, and its incidence increases with an aging population, as this disease is most commonly found in adults [1]. In the past 5 years, the cure rate was 35–40% for AML patients under 60 years old and 5–15% for patients older than 60. The elderly, who are unable to withstand intensive chemotherapy, have an average survival of 5–10 months [2]. Despite improving our understanding of AML, the disease still has poor outcomes due to high disease- and treatment-related mortality.

Forty years ago, the combined injection of cytarabine and anthracycline was introduced as the first standard treatment for AML [3, 4]. Since then, many chemotherapy regimens have improved outcomes for some AML patients [5]. However, the effectiveness of traditional chemotherapy may have hit a ceiling for treating AML, especially for older patients and those who either tend to relapse or have intermediate- or high-risk factors associated with AML [6]. In addition, allogeneic hematopoietic stem cell transplantation (allo-HSCT) has been the most successful immunotherapy for AML over the past decade, especially with the advances made in using alternative donors [7–9]. Unfortunately, older and less fit patients are poor candidates for allogeneic HSCT due to significant toxicity and a high relapse rate [10]. The limited success and high toxicity of the currently available strategies indicate an urgent need for new therapeutics. It is possible that the infusion of allogeneic chimeric antigen receptor (CAR) T cells could enhance the efficacy of allogeneic HSCT [11]. This possibility is supported by recent evidence that a child with acute lymphoblastic leukemia (ALL) at the Children’s Hospital of Philadelphia relapsed after a cord blood transplant and then received infusions of CTL019 CAR T cells, resulting in a remission of leukemia without graft-versus-host disease (GVHD) [12]. In addition, another recent study showed that the treatment of allogeneic CAR T cells is beneficial for patients with relapsed B cell malignancies after allo-HSCT with low toxicities and complications [13].

**Fig. 1:**
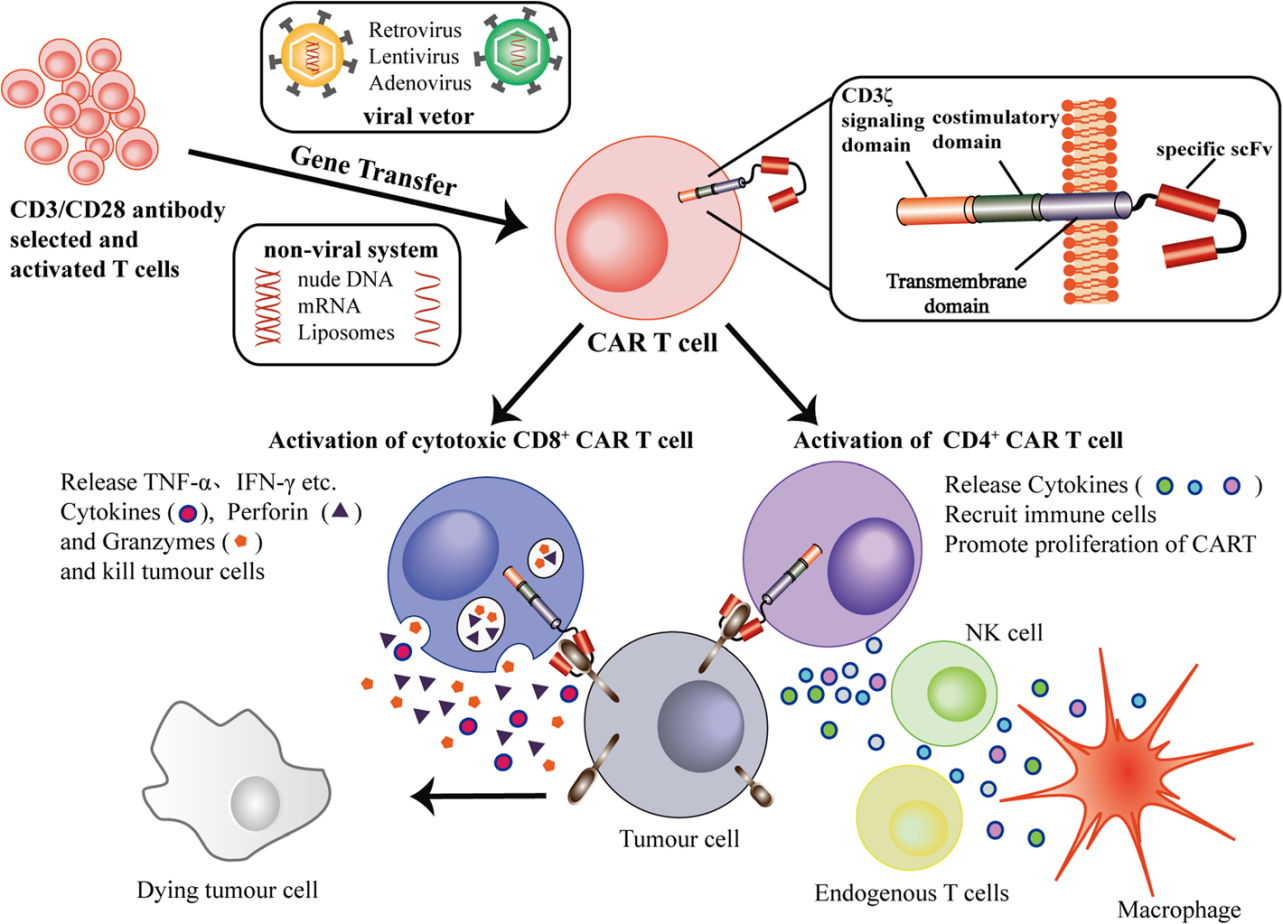
The process of CAR T cell activation and killing of tumor targets. T cells are collected from patients and then selected and activated by a CD3/CD28 antibody. The CAR gene was transferred by viral vectors or non-viral systems. When CAR recognizes its antigen on tumor cells and binds it, the intracellular signaling domains within the CAR produce a series of signal transduction cascades, and then, the CAR T cell is activated. The activation of cytotoxic CD8+ CAR T cells releases TNF-α, INF-γ, granzyme and perforin, which directly kill tumor cells. In addition, tumor killing can also be mediated by the activation of other components of the immune system through cytokines released by CD4+ CAR T cells. Notably, the defining characteristic of CAR T cells is that they produce long-term memory CAR T cells after the initial activation, which will be of great benefit to the long-term tumor eradication and the prevention of tumor relapse

Therefore, the CAR-expressing T cell technology, which has been successfully implemented in treating acute lymphoblastic leukemia (ALL), has been considered a promising immunological approach for the treatment of AML [12, 14–19]. This new type of targeted immunotherapy merges the exquisite targeting specificity of monoclonal antibodies with the potent cytotoxicity and long-term persistence provided by cytotoxic T cells. CAR is an artificial antigen receptor that mediates antibody-targeted recognition. The binding between CAR and its antigen on tumor cells triggers a signal transduction cascade through signaling domains and then activates T cells to kill the target directly or through other components of the immune system (Fig. 1) [20]. At the beginning of the in vitro expansion stage, CAR can be transferred to the patient’s selected T cells using either viral vectors or non-viral approaches [21]. The viral vectors include retroviruses (including lentivirus), adenovirus and adeno-associated virus. Among them, γ-retroviral and lentiviral vectors have been the most useful carriers for long-term gene expression because of their ability to integrate into the host genome and their low intrinsic immunogenicity [22, 23]. In contrast to γ-retroviral vectors, lentiviral vectors can deliver larger DNA sequences and integrate into non-dividing cells, which are less susceptible to silencing by host restriction factors [24]. Lentiviral vectors are more commonly used in clinical trials because of their safer integration site profile [25]. Non-viral systems, including nude DNA, mRNA, liposomes, etc., are very effective in gene delivery because of their higher efficiency, non-infectiousness, unlimited carrier capacity, controlled chemical constitution and generous production. For instance, mRNA electroporation in clinical trials induced the transient expression of CAR for approximately one week and prevented the potential toxicity of CRS [11].

**Fig. 2:**
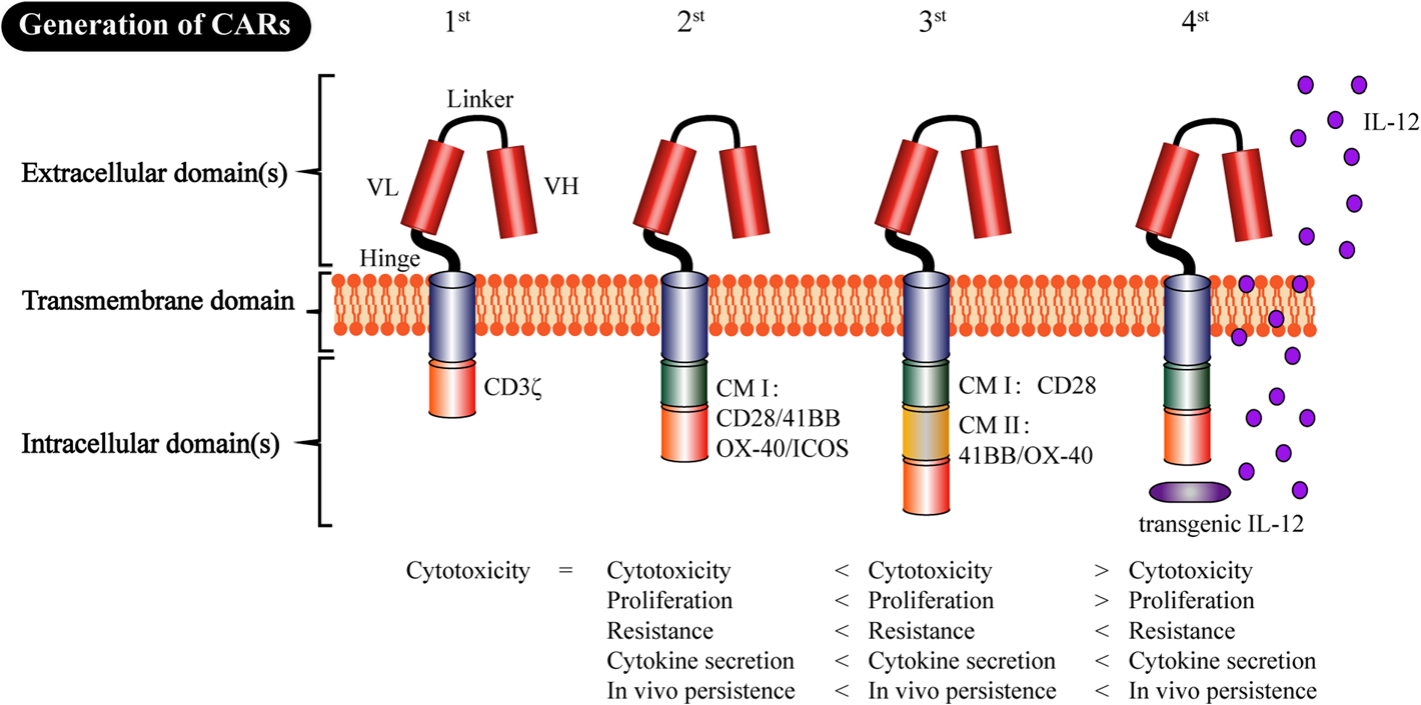
The four generations of CAR production. The extracellular domain of CAR includes a single chain variable fragment (scFv) (H (heavy) and L (light) chain) that is spliced by a linker. A hinge (e.g., hinge region of human immunoglobulin D molecule) ensures flexibility and connects to the transmembrane domain (TM). The TM is routinely the constant region of the human G immunoglobulin, whereas the intracellular domain includes only the CD3ζ signaling domain known as the “1st-generation CAR”. Subsequently, to augment T cell persistence and proliferation [28], CD3ζ as well as the costimulatory endo-domains 41BB- or CD28-signaling domains were incorporated into the “2nd-generation CAR”. The intracellular domain includes CD3ζ plus two costimulatory domains 41BB- and CD28-signaling domains that were included in the “3rd-generation CARs” [31, 32]. So-called TRUCK T cells are known as the “4th-generation CAR”, which is additionally modified with an inducible expression cassette for a transgenic protein

The adoptive cell therapy of CAR-expressing T cells is a new but promising approach in the field of cancer immunotherapy. The development of CAR T cells can be divided into four generations based on the different characteristics of intracellular domains (Fig. 2). The CAR prototype consists of an extracellular domain that serves as the targeting moiety (which is usually a single chain variable fragment (scFv) formed from a monoclonal antibody, mAb), a transmembrane domain, and an intracellular signaling domain(s) [26]. CARs with the typical structure of “scFv-spacer-CD3z” design are called “1st-generation CARs”. In contrast to the T cell receptor (TCR), “1st-generation CAR” recognizes the targets independent of major histocompatibility complex (MHC) restriction, therefore making it highly specific for various surface antigens on tumor cells [27]. However, further research has gradually shown that “1st-generation CARs” exhibit the problems of deficient secreted cytokines, inadequate proliferation, and low persistence in vivo. To overcome these weaknesses, CD3ζ as well as co-stimulatory signaling domains, such as 41BB and CD28, were incorporated into the intracellular domain to form the so-called “2nd-generation CAR” [28–30]. The added co-stimulatory signal can help complete the activation of T cells and avoid apoptosis by promoting the IL-2 synthesis. The CD28ζ CAR T cells primarily caused constitutive stimulation, proliferation and growth. In contrast, the 41BBζ CAR T cells induced early exhaustion, thereby limiting the antitumour efficacy [21]. Correspondingly, the CD3ζ plus two co-stimulatory signaling domains, 41BB- and CD28, were introduced into the intracellular domain of “3rd-generation CARs” to augment cytokine production and cancer-killing ability [31, 32]. Unfortunately, the latest studies have shown that the “3rd-generation CARs” did not produce more desirable outcomes compared with the “2nd-generation CARs”. Because the stronger stimulation may produce potential side effects, such as cytokine release syndrome (CRS), further studies should be performed to explore the safety of “3rd-generation CARs”. Notably, recent studies have indicated that CAR reached its limit when targeting tumors with a remarkable phenotypic heterogeneity. Subsequently, the “4th-generation CARs”, i.e., so-called TRUCK T cells, were proposed, which are formed by an additional modification with an inducible expression cassette for a transgenic protein. For example, a cytokine such as IL-12 can be released by CAR T cells to modulate the T cell response, which can help keep the local therapeutic concentration and avoid systemic toxicity by releasing a variety of therapeutic proteins [27]. We anticipate that the future new generations of CAR will overcome some of the corresponding problems of CAR T cell therapy.

Translating the success of CAR T cell therapy to other malignancies that have an unmet medical need, such as AML, is currently underway. For instance, CAR T cell therapy has been developed in patients with AML, as many detailed studies have been published. This review summarizes the recent application of CAR T cell therapy in AML and focuses on AML-associated cell surface antigens that could be potential target candidates for CAR T cell therapy. Finally, we discuss the common issues of CAR T cell therapy in AML and summarize the strategies of building CAR T cells with improved safety and availability.

### The application of CAR-modified T cells in AML

**Fig. 3:**
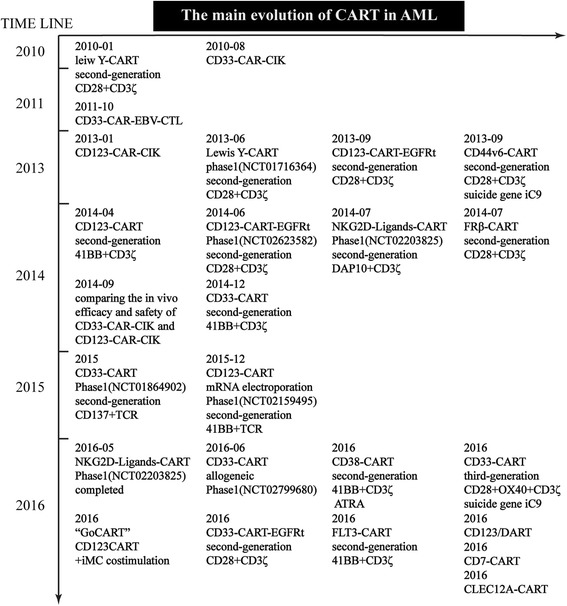
The main evolution of CAR T cell therapy for treating AML. CIK, cytokine induced killer; EBV-CTL, human Epstein Barr Virus-cytotoxic lymphocyte; EGFRt, a tag derived from the epidermal growth factor receptor, is the antigen of the clinically available antibody cetuximab; DAP10, a type of natural adaptive protein, provides a costimulatory signal similar to that of CD28 [113]; ATRA, all-trans retinoic acid, a drug that up-regulates the expression of the target antigen, resulting in improved anti-leukemia activity; GoCART, a structure comprising a proliferation-deficient first generation CAR and a ligand-dependent activation switch (e.g., iMC) that efficiently eradicates CD123+ AML cells when co-stimulated with systemic rimiducid administration. iMC, inducible MyD88/CD40 is a ligand (rimiducid)-dependent costimulatory switch [114]

Despite the enormous challenge of developing CAR T cells for multiple diseases, several potential CAR T cell targets have been actively explored in preclinical studies and clinical trials over the past decade (Fig. 3). From the evolution of CAR T cell therapy for treating AML, it may be observed that we have gradually shifted the focus of our research on creating safer and more effective CAR-modified T cells for the past two years using genomic editing.

#### Lewis Y antigen

One of the remarkable advantages of CAR T cell therapy is the ability to recognize a broad variety of targets such as non-protein antigens. The Lewis Y antigen (LeY) is an example of this situation; LeY is an oligosaccharide that is overexpressed on many epithelial cancers and hematological malignancies (including AML) [33, 34] but has limited expression on normal healthy tissues [35, 36]. The LeY - CAR T cell trial was the first CART therapy clinical trial targeting AML (ClinicalTrials.gov number, NCT01716364), evaluating the effect of an autologous second-generation anti-LeY CAR T cell therapy in 4 patients with relapsed AML. Following fludarabine preconditioning, the patients were administered up to 1.3 × 10^9^ of total T cells (14–38% CAR T cells). The results showed that two patients achieved protracted remission, one patient achieved cytogenetic remission, and the fourth patient with active leukemia presented a reduction in peripheral blood (PB) blasts. Incredibly, no grade 3 or 4 toxicity was observed. The most notable finding from this study was the lack of toxicity and the durable in vivo persistence after infusion [37]. In addition, LeY is the first antigen that was successfully implemented in CAR T cell therapy to target AML. Retroviral transduction of anti-LeY-CD28ζ into CAR T cells has exhibited potent cytotoxicity against LeY^+^ epithelial tumor cell lines in vitro and animal models in vivo without affecting normal tissues [38].

#### CD44v6

The hyaluronate receptor CD44 is a type I transmembrane glycoprotein commonly used as a marker to identify cancer stem/initiating cells. CD44 variant domain 6 (CD44v6) is a CD44 variant isoform expressed in AML [39] and multiple myeloma (MM) [40], correlating with a poor prognosis. Importantly, CD44v6 is absent in hematopoietic stem cells (HSCs) and expressed at low levels on normal cells, which may provide a therapeutic window. The Italy San Raffaele Scientific Institute designed a second-generation CD28-CD3ζ CAR and derived the scFv from a mutated sequence of the humanized CD44v6-specific mAb (bivatuzumab). This CAR exerted a significantly positive effect in targeting cancer cells in vitro and in vivo. However, these anti-CD44v6-CD28ζ CAR T cells caused an unexpected and dose-limiting toxicity (DLT), monocytopenia. Subsequently, this group focused their attention on co-expressing clinical-grade suicide genes [41, 42] to control these adverse events [43].

#### NKG2D ligand

Natural killer group 2D (NKG2D) ligands contain six members of the UL16-binding protein, or the retinoic acid early transcript (ULBP/RAET) family and two members of the MHC class I-related chain (MIC) family [44], all of which are either absent or minimally expressed on healthy tissues but widely expressed on numerous malignancies (including ovarian cancer [45] and AML [46]). Several different variants of NKG2D-directed CAR have been developed and tested for their cytotoxicity and the ability of achieving complete remissions [47]. From April 2015 to July 2016, a phase I (ClinicalTrials.gov number, NCT02203825) dose-escalation study was performed to establish the feasibility and safety of NKG2D-DAP10-CD3ζ CAR T cells (CM-CS1 T cells) in treating AML and was completed ahead of schedule. A total of 11 subjects were infused with 1 × 10^6^ to 3 × 10^9^ (8 cohorts) of CM-CS1 T cells based on a 3 + 3 design. The results showed that 9 subjects treated in the first 3 cohorts completed their 28-day evaluation period without any DLTs. It is worth mentioning that there was no case of cell-related neurotoxicity, cytokine release syndrome (CRS), autoimmunity or CAR T cell-related death during treatment [48].

#### Folate receptor β

The folate receptor β (FRβ) is a member of the folate-binding protein receptors family, which is primarily expressed on myeloid-lineage hematopoietic cells and frequently up-regulated in AML blasts (~70%) [49, 50]. Preclinical models using anti-FRβ-CD28ζ CAR T cells presented potent and targeted killing of leukemia cells while preserving healthy CD34+ cells. Interestingly, the investigators also used all-trans retinoic acid (ATRA), an FDA-approved drug for subclass M3 AML [51, 52], to up-regulate the target antigen, which led to an improved anti-leukemia activity [53]. This general concept of increasing antigen expression on diseased tissue to improve the potency of the CAR T cell agent is very likely to be further explored in follow-up studies.

#### CD38

CD38, also known as cyclic ADP ribose hydrolase, is a glycoprotein expressed on the surface of many immune cells. Previous studies have shown that CD38 is expressed on the majority of AML blasts but not healthy human hematopoietic stem cells (HSCs) [54, 55]. Accordingly, one research group has focused on CD38 as a candidate therapeutic target and developed an anti-CD38-41BBζ CAR. Remarkably, studies involving this CAR revealed another example of ATRA-enhanced cytotoxicity on AML cells regarding enhanced CD38 expression [56]. Therefore, these results may provide a new paradigm for pharmacologically inducible immunotherapy that combines ATRA and CAR T cell therapy to treat AML.

#### FLT-3

Fms-like tyrosine kinase 3 (FLT-3), also known as CD135, is a cytokine receptor belonging to the class III receptor tyrosine kinases. The FLT3 gene is one of the most commonly mutated genes in AML, with internal tandem duplications of FLT3 (FLT3-ITD) as the most frequent mutation (25%) associated with AML. In a recent study, researchers generated anti-FLT3-41BBζ CAR T cells, which demonstrated potent anti-AML activity in vitro and in vivo. Notably, compared with anti-CD33 CAR T cells, anti-FLT3 CAR T cells indicated a lower hematological toxicity [57].

#### CD7

CD7 is an NK and T cell marker that is highly expressed in 30% of AML cases. Its expression is associated with a worse prognosis and chemoresistance [58, 59]. CD7-directed CAR T cells have been created and exhibited potent cytotoxicity against T-ALL and AML cell lines as well as against primary AML blasts, but there was no observed toxicity against normal myeloid progenitors [60]. This finding indicates that CD7 is a potential target for AML that should be further explored in future studies.

#### CD33

CD33 is a transmembrane receptor of the SIGLEC family and is expressed in approximately 90% of AML patients as well as on AML stem cells [61, 62]. Because CD33 is a notable and promising myeloid-specific target, many groups have independently designed CD33-directed CAR T cells (in Fig. 2) and reported potent anti-leukemia outcomes using AML tumor cells and primary xenograft models [63–68]. Importantly, a phase I study at the Chinese PLA General Hospital (ClinicalTrials.gov number, NCT01864902) used lentivirally transduced anti-CD33-41BBζ CAR T cells delivered in escalating fractions to a single patient with refractory AML, which resulted in a transient response [67]. However, as CD33 is expressed in healthy myeloid cells and other tissues [69–71], the toxicity that occurs following CD33-directed CAR T cell infusion must be well controlled before further evaluation in clinical trials. One research group proposed a novel solution to this problem by removing CD33 from normal hematopoietic stem progenitor cells (HSPCs) using genomic editing during CD33-mediated CAR T cell treatment of AML, as CD33 is not essential to hematopoietic differentiation, and a lack of CD33 in myeloid progeny does not cause any visible functional changes [72]. Overall, recent studies were committed to reducing the toxicity of CD33-specific CAR T cells and proposed many strategies, which will be further described in detail below.

#### CD123

As the transmembrane alpha chain of the interleukin-3 receptor, CD123 is widely expressed in the majority of AML blasts but presents low expression levels on normal hematopoietic cells [73–77]. Both anti-CD123-CD28ζ CAR and anti-CD123-41BBζ CAR T cells have demonstrated potent leukemia killing ability in vitro and in vivo but produced incongruous results regarding their myeloablative effect on healthy CD123^+^ cells [78, 79]. In addition, two phase I trials (ClinicalTrials.gov number, NCT02159495, NCT02623582) for CD123-directed CAR T cell therapy are currently underway to validate the effect and safety profiles. Subsequently, one group generated a novel anti-CD123-CD28-CD137-CD27-CD3ζ-iCasp9 CAR (4SCAR123) that exhibited potent cytotoxicity against AML in vitro and then infused 4SCAR123 into a 47-year-old male patient with AML-M2. The patient exhibited a rapid response consistent with a controllable CRS and achieved partial remission within 20 days without any off-target cytotoxicities [80]. One significant concern is that CD123-directed CAR T cells could irreversibly increase the myeloablative impact on normal hematopoiesis. Some strategies have been proposed to develop safer CD123-directed CAR T cells, one of which involves using the irreversible myeloablation of CD123-directed CAR T cells in conjunction with allogenic HSCT, such as the chemotherapy preconditioning prior allo-HSCT, to reduce the risk of AML relapse and pave the way to further explore CAR T cell combination therapies [78].

#### CLEC12A

CLEC12A (also known as CLL1) has been previously described as selectively overexpressed in leukemia stem cells (LSCs). One group confirmed that CLEC12A is heterogeneously expressed on AML blasts and overexpressed on AML LSCs. Lentivirally transduced anti-CLEC12A-41BBζ CAR T cells can successfully target CLEC12A^+^ cells, which are resistant to chemotherapy. Hence, anti-CLEC12A CAR T cells can potentially be used as a consolidation regimen after induction chemotherapy to eradicate LSC and minimal residual disease (MRD) in AML [81].

### AML-related surface antigens as candidates for CAR therapies

Due to its potent and durable anti-tumor activity, CAR T cell therapy has been recently regarded as a promising curative therapy against B-lineage malignancies. The reason for these positive results is that CD19 is an ideal target for B-cell malignancies [65]. As is well known, new tumor-related antigens may arise following somatic mutations in the dividing tumor cells, which can serve as valuable therapeutic targets. These antigens are classified as tumor-specific antigens and mutation-causing over-expression antigens [82]. CD19 is a unique tumor-specific antigen expressed on the tumor cells of B-lineage malignancies but not on normal cells. Unfortunately, truly AML-specific surface antigens have not been identified to date. Most of the antigens currently studied are mutation-causing over-expression antigens, which result in fatal “on-target/off-tumor toxicity” of CAR T cell treatments because of the expression of these antigens on normal tissue. Therefore, one prerequisite for developing clinically effective CAR therapies is the confirmation of specific AML-associated surface targets. Theoretically, these antigens should meet the following specific requirements [83]: 1) a confirmed AML surface antigen; 2) expressed on as few normal tissues as possible; 3) expressed in an adequately large percentage of AML patients; 4) homogenously expressed on the tumor cells of a given patient; and 5) exerts an essential function in the pathophysiology and/or biology of AML [84].

In addition to the above-mentioned targets used in CAR T cell therapy to treat AML, several other surface molecules, which are listed in Table 1, have been identified and may be useful for directing the future exploration of CAR T cells in AML based on their distribution in normal tissue and specific involvement in potential toxicity [84].

**Table 1:** Cell surface antigens expressed on AML compared with HSC

Antigen	AML expression	Function	Normal tissue expression	Comment	Reference
CD44	100% (samples)	Mediates cell adhesion and can transduce signals	Ubiquitously expressed with many alternatively spliced isoforms	Cancer stem cell marker on several solid tumors	[123]
CD45RA	>90% (samples)	Regulates a variety of cellular processes including cell growth, differentiation, mitotic cycle, and oncogenic transformation	naive T cells; CD34^+^CD38^+^ normal progenitors	A specific marker for leukemia stem cell subpopulations in AML	[124]
CLL-1 (CLEC12A, DCAL-2, MICL)	≈95% (samples)	ND	Restricted to hematopoietic cells of myeloid lineage	Expression may identify minimal residual disease and predict relapse	[81, 125, 126]
CD96	66% (samples)	May have a function in NK cell adhesion and/or activation	Resting and activated T cells and NK cells, possibly intestinal epithelium	Expressed on only 5% of CD34^+^CD38^−^CD90^+^ cells in bone marrow	[127]
CD47	100% (samples)	Binds SIRPa and inhibits phagocytosis	Widely expressed at low levels	Differential expression facilitated prospective separation of residual normal HSC from LSC	[128, 129]
CD32	34% (samples)	Fc-g receptor 2 (FCGR2)	Restricted to hematopoietic cells	Not expressed on functional HSC	[130]
CD25	25% (samples)	High-affinity IL-2 receptor (IL2RA)	Restricted to hematopoietic cells	Not expressed on functional HSC	[130]
TIM-3 (HAVCR2)	most AML types (except for M3)	An important regulator of Th1 cell immunity and tolerance induction	Not expressed in CD34^+^CD38^−^ normal HSCs or the majority of CD34^+^CD38^+^ normal progenitors	An immune checkpoint, also a Th1-specific cell surface protein that regulates macrophage activation	[131–133]

*ND* not detected, *NK* natural killer, *HSC* hematopoietic stem cell, *LSC* leukemia stem cell, *SIRPa* signal regulatory protein-a

Our group currently select optimal AML targets for future study based on the safe and effective results of matured antibody technology depicted in Table 2. In addition, our group allowed that the new trend to target the LSCs rather than tumor cells for CAR T cell therapy may lead to better cancer treatment. Because the so-called LSCs, which are not effectively eliminated by current treatments, retain extensive self-renewal and tumourigenic potential that induces tumor proliferation and progression, it has been long proposed that AML has a high rate of relapse [85]. As previously mentioned, CD123 is a typical LSC target in AML, and it has been reported that CD123-CAR T cells may be a promising tool as a chemotherapy-free myeloablative conditioning regimen for HSCT, which is particularly critical to avoid relapse [79]. As shown in Table 1, CD47 is overexpressed on LSCs and can be detected in almost all AML samples, and its expression is often associated with worse outcomes [86]. AML LSCs escape macrophage phagocytosis by the recognition between CD47 on the LSCs and extracellular region of signal regulatory protein alpha (SIRPα) on the macrophages [87]. By contrast, CD47 is faintly expressed in most normal tissues [84]. These findings make CD47 an ideal marker of AML LSCs. T-cell immunoglobulin mucin-3 (TIM-3) is another ideal marker of AML LSCs and is highly expressed in LSCs in most types of AML (except for M3) but is not expressed in normal LSCs [88]. TIM-3 plays an important role in the viability, proliferation, and differentiation of AML LSCs [89], as well as in the exhaustion of CD8+ T cells. Several recent studies have shown that AML relapse after CAR T cell therapy is directly associated with the significant up-regulation of TIM-3 receptors on T cells. TIM-3 pathways are also involved in the exhaustion of CAR T cells and the dysfunction of AML [90, 91]. This pathway is worth further exploration as a potential target in the clinical setting.

**Table 2:** AML-related surface molecules as potential targets for CAR therapies

Antigen	Antibody clone	Efficacy in treatment model	Effect on normal	References
CD47	B6H12 (mouse IgG1)	Treatment initiated 8–12 weeks post transplantation: decrease AML in 3/3 samples (8/8 mice) with clearance of the bone marrow in 3/8 mice	No effect on in vitro phagocytosis of CD34^+^ normal bone marrow progenitors	[128]
	Hu5F9 (Human IgG4)	Completely eradicated human AML in vivo, leading to long-term disease-free survival of patient-derived xenografts	Safely administered intravenously at doses by toxicokinetic studies in non-human primates	[134]
TIM3	ATIK2a (human IgG2b)	Effective in killing TIM-3 expressing cell lines by its CDC and ADCC activities; In vivo xenogeneic transplantation efficiently eradicated AML LSCs	No effect on cord blood or bone marrow engrafted mice	[133]

*AML* acute myeloid leukemia, *CDC* complement dependent cytotoxicity, *ADCC* antibody-dependent cell-mediated cytotoxicity, *LSC* leukemia stem cell

### The challenges and corresponding strategies of CAR T cell therapy in treating AML

**Fig. 4:**
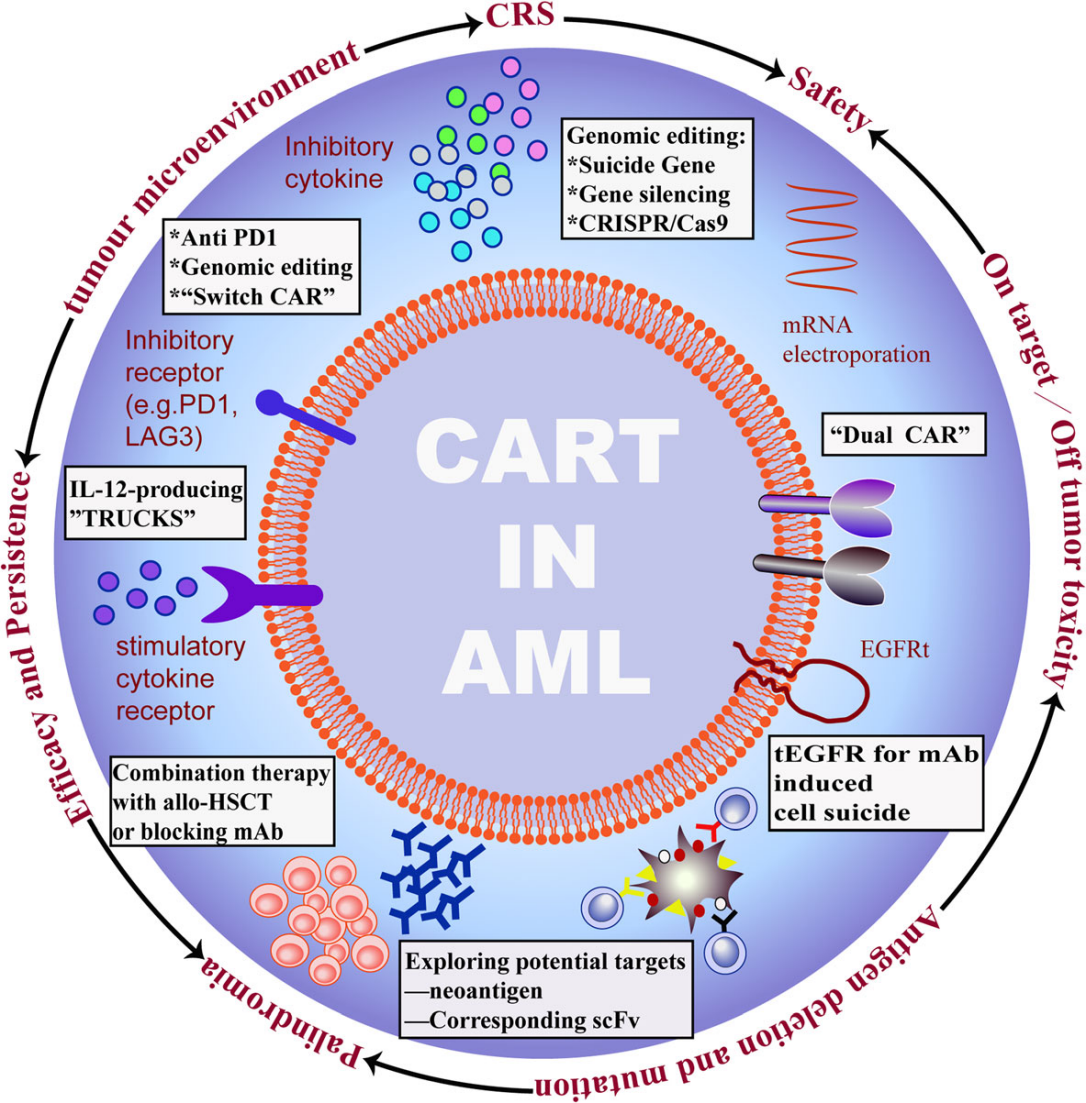
Creating a better CAR-expressing T cell. mAb, antibody monoclonal antibody; scFv, single chain antibody fragment; allo-HSCT, allogenic haemopoietic stem cell transplantation; iCasp9, inducible caspase 9; IL12, interleukin-12; LAG3, lymphocyte activating 3; mRNA, messenger ribonucleic acid; PD1, programmed death 1; EGFRt, truncated epidermal growth factor receptor; TRUCKS, T cells redirected for universal cytokine-mediated eliminating antigen-negative cancer cells

CAR-redirected T cells are an emerging powerful tool for treating patients with cancer, with an especially high rate of long-term complete remission achieved by CAR T cell treatments in relapsed/refractory CD19^+^ ALL patients [17, 19, 92]. Over the past few years, several groups have concertedly focused on translating CAR T cell therapy to AML, and they have demonstrated that CAR T cells can eradicate AML in both preclinical and clinical trials. Thus, the efficacy of anti-AML CAR T cells appears to be equivalent to that of anti-ALL CAR T cells. Nevertheless, critical questions remain in this field. Here, we will outline the challenges of CAR T cell therapies when applied to AML, and focus on discussing the available and potentially feasible strategies to optimize the efficacy and safety of CAR T cell therapy (Fig. 4).

#### Cytokine release syndrome

When CAR T cells exert a clinical effect, persistence and proliferation are required; however, these activities may also cause significant toxicity. The most common and harmful toxicity is cytokine release syndrome (CRS), a rapid and evident inflammatory systemic response caused by dramatic increases in many inflammatory cytokines (e.g., soluble IL-2R, IL-6 levels, ferritin, C-reactive protein (CRP), etc.) that occur with the in vivo activation and exponential proliferation of CAR T cells. [93]

As previously reported by Wang et al., one AML patient treated with approximately 4 × 10^8^ anti-CD33 CAR T cells experienced CRS [67]. Another group submitted an abstract that described a single patient treated with anti-CD123 CAR T cells who showing severe CRS in the absence of overt off-target cytotoxicity [94].

Many studies have indicated that IL-6 is a central mediator of CRS-related toxicity [93]. Furthermore, several clinical studies have proved that the combined administration of tocilizumab, an anti-IL-6R antagonist, and systemic corticosteroids showed successful and rapid relief of CRS following CAR T cell infusions [12]. The clinical treatment algorithm for CRS has been well reviewed; please refer to reference 95 [95].

**Fig. 5:**
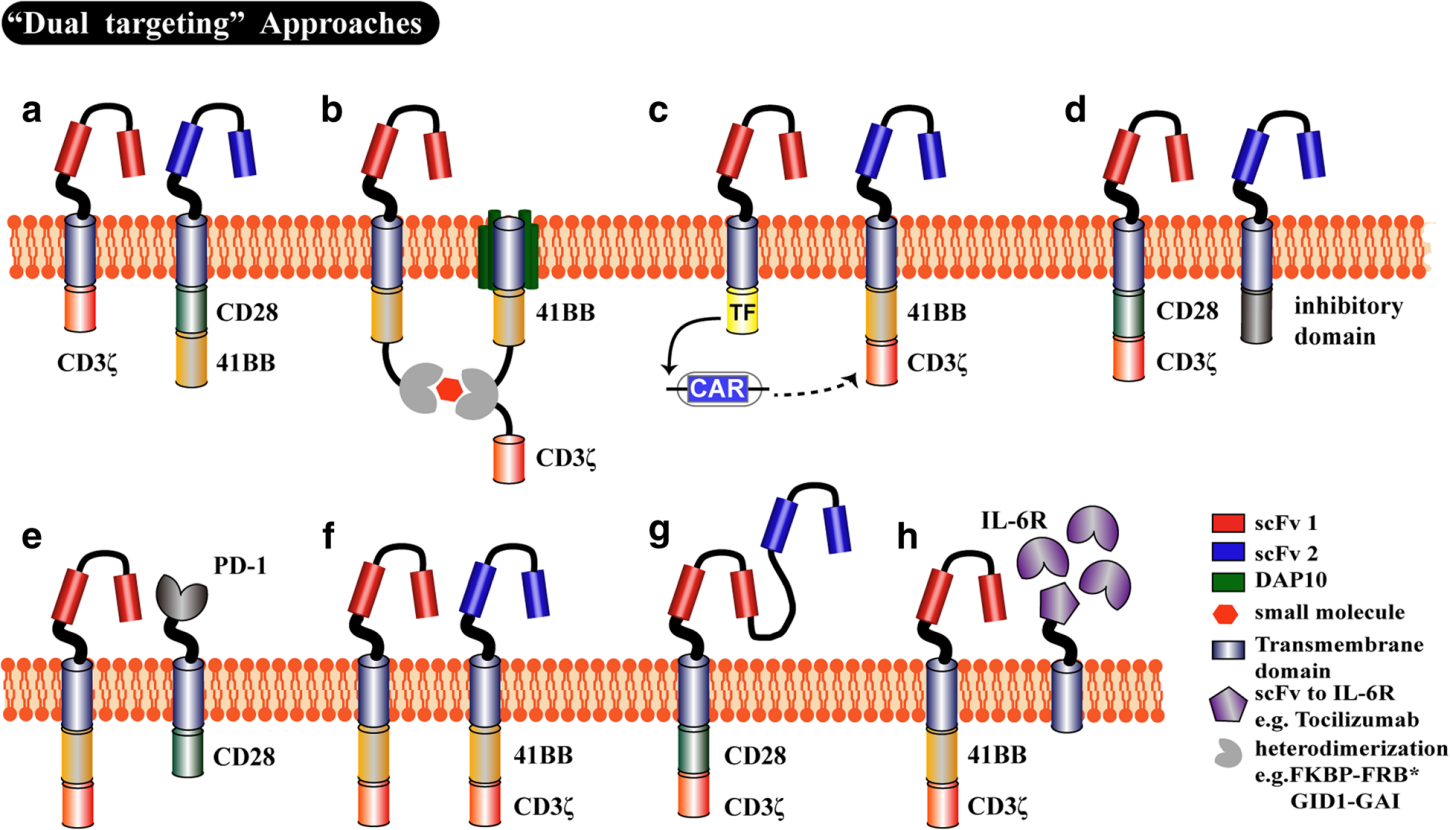
Different types of “Dual targeting” approaches. **a** The CD3ζ and costimulatory domains are separated in individual molecules targeting two diverse tumor antigens, an event known as trans-signaling CARs. These proteins will be activated when both antigens are identified [115–117]. **b** The “ON-switch” CAR T cell requires a small molecule drug to activate an “ON-switch” such that the engaging antigen and intracellular signaling domain will be connected [118]. **c** The mechanism of “notch CAR” recognizes combinatorial antigens by using a synthetic Notch receptor for one antigen that drives the inducible expression of the CAR target to a second antigen; this system requires a tumor cell to express both antigens before recognition by the CAR T cells [119]. **d** An inhibitory CAR replaces the CD28- CD3ζ chain with an inhibitory domain, which limits the excess activation signal from other CARs [120, 121]. **e** “PD1CD28” switch CAR T cells express a switch receptor construct comprising the PD1 extracellular domain and the CD28 costimulatory domain; this allows PD-L1 binding to enhance CAR T cell cytokine secretion and proliferation [122]. **f** Dual-signaling CAR, T cells are respectively modified by two distinct CAR molecules with two different scFvs and the same intracellular signaling domain [108, 109]. **g** Tandem CARs comprise two different linked scFvs to allow for targeting of two different antigens using a single construct [112]. **h** This is a hypothetical strategy to reduce CRS and was inspired by the “PD1CD28” switch CAR T cells

Strategies of further optimizing the treatment algorithms for CRS are currently under investigation (ClinicalTrials.gov number, NCT02906371), and gene-editing technology could be applied to CAR T cells to avoid CRS-related toxicities. For example, either gene silencing or the CRISPR/Cas9 system can be used to disturb IL-6 and other CRS-related cytokines in T cells prior to transduction with CARs. Additionally, T cells could simultaneously express a corresponding scFv specific to the IL-6 receptor such as tocilizumab as well as CARs in order to block the IL-6 receptors actively avoiding CRS (Fig. 5h).

In all, the mechanisms by which CAR T cells cause CRS are varied and poorly understood. How to effectively control the CRS toxicity of CAR T cells is one of the most important challenges for improving the field of CAR T cell therapies overall.

#### On-target/off-tumor toxicity

Because on-target/off-tumor toxicity results from the expression of tumor-associated antigens (TAAs) on normal tissue, minimizing the risk of toxicity is critical in the successful implementation of CAR T cell therapy. The first step in this process is to select more specific AML-associated surface targets, as mentioned above. However, it is highly difficult to identify surface antigens that are uniquely expressed on malignant myeloid tumors. There are many reports regarding insignificant myelosuppression caused by CAR T cells in preclinical models of AML. In addition, one AML patient enrolled in NCT01864902 experienced moderate hepatotoxicity and a transient reduction in marrow blasts following infusion with anti-CD33 CAR T cells [67]. Another clinical trial with anti-LeY CARs in AML did not reveal any major off-target toxicities [37].

In consideration of the serious consequences of the “on-target/off-tumor” toxicities reported in other clinical cases [96, 97], we should prepare corresponding strategies to address the “on-target/off-tumor” effects that may arise at any time.

#### mRNA electroporation

The expression of CARs using mRNA electroporation of T cells ensures the gradual loss of surface CAR expression as T cells divide, which may be a useful strategy for determining the potential toxicity of novel constructs. One group transiently expressed an mRNA CAR construct targeting CD33 to avoid prolonged toxicity [65], whereas another clinical study is currently ongoing in which T cells expressing anti-CD123 CARs via mRNA electroporation were infused into patients with AML (ClinicalTrials.gov number, NCT02623582) to evaluate efficacy and safety.

#### Suicide gene applications

A suicide gene is a genetically encoded molecule that allows for the selective destruction of adoptively transferred cells. The addition of a suicide gene to cellular therapeutic products can lead to the selective ablation of gene-modified cells, which can mitigate or prevent collateral damage to contiguous cells and/or tissues [32]. This approach may be useful in abrogating the on-target and off-tumor toxicities of CAR-directed T cells. The inducible Caspase9 (iC9) suicide gene comprises a drug-binding domain cloned in frame with human Caspase9. Upon the exogenous administration of a non-therapeutic small molecule chemical inducer of dimerization (CID), iC9 dimerizes and induces apoptosis of the transduced cells within hours. CD44v6-, CD33-, and CD123-directed CAR T cells all contain an iC9 suicide gene as a tool for controlling the adverse events, which has been tested in preclinical research [37, 68, 80].

#### “Kill switch”—EGFRt

A “kill switch” is based on a tag derived from the epidermal growth factor receptor (EGFRt) that retains the epitope recognized by the commercially available FDA-approved mAb cetuximab [98]. Anti-CD33- and anti-CD123-CD28ζ-EGFRt cells have been designed that can be eliminated by cetuximab if either CRS or any on-target/off-tumor toxicities are observed [99–101].

#### Dual-targeting strategies

When off-tumor toxicity is observed, these above strategies could enhance the ability of either ameliorating or abrogating these deleterious effects. Therefore, the inclusion of up-front safeguards is in an urgent need to prevent off-target toxicity in healthy tissues. Specific novel strategies are described in Fig. 5a-d, but future studies are required to expand on these ideas.

### Relapse

Despite the scarcity of clinical cases regarding AML relapse after CAR T cell therapy, several preclinical studies have been performed to explore the reasons for the relapse. The corresponding strategies to address this issue have also been proposed.

#### Reduced efficacy and LSCs

Relapse is primarily caused by the lack of effectiveness of CAR T cells, which can be attributed to two factors: the immunosuppressive microenvironment and LSCs. To address the first issue, one approach is the use of so-called “TRUCK cells”, which can induce IL-12 release and activate innate immune cells to the targeted tumor and thus eliminate cancer cells not recognized by CAR T cells [27]. This strategy can enhance the efficacy of CAR T cell therapy, thereby eliminating cancer cells and preventing tumor relapse caused by the residual cancer cells. To address the second issue regarding LSCs, the best solution is to identify the optimal markers for AML LSCs applied to CAR, which we have discussed in detail above.

#### Immune checkpoint

Inhibitory receptors/pathways, such as the PD-1 and TIM-3 pathways, induce the dysfunction and exhaustion of CAR T cells in AML and are also the mechanism of immune escape. Recently, several studies have indicated that there is a significantly higher expression of PD-1 and TIM-3 on T cells in relapsed AML samples compared with that seen in remittent or healthy donors [91, 102, 103]. Gene-editing technology could allow for the permanent disruption of negative signaling pathways [104]. Combined approaches using blocking antibodies may also interrupt this interaction, thus leading to the increased CAR T cell-induced cytotoxicity [103]. The latest technology is the use of switch receptors that incorporate a segment of the PD-1 receptor into the CAR construct (Fig. 5e), thereby inducing PD-L1 expression within the tumor microenvironment (TME) to augment the cytokine secretion, proliferation and granzyme expression of CAR T cells, improving tumor therapy [105].

#### Antigen escape

A typical clinical case we observed is when an AML patient experiences relapse following CD33-CAR T cell treatment because leukaemic cells can selectively proliferate AML cells with low CD33 expression to evade the identification by CAR T cells [67]. The antigen escape-caused relapse involves multiple mechanisms. With the exception of the above-mentioned case, antigen loss on the tumor surface and deleterious mutations of antigens recognized by CAR-T cells have been observed in ALL clinical cases [106]. One clinical scenario is that CD19 is still present but cannot be detected and recognized by anti-CD19 CAR-T cells as its cell surface fragment is absent because of a deleterious mutation or alternative splicing [107]. A new strategy to address the antigen escape-caused relapse involves designing CAR T cells able to be activated by multiple antigens synchronously. Other dual-targeted CAR-T cells have been investigated in preclinical studies. One is known as dual-signaling CAR T cells (Fig. 5f), which are modified by two distinct CAR molecules with different binding domains [108, 109]. Another type is the so-called Tan-CAR T cells (Fig. 5g), which are modified by one CAR molecule with two different binding domains in tandem [110–112]. Both dual-signaling CAR and TanCAR can control antigen escape-caused relapse because a single antigen can trigger robust anti-tumor activity. Currently, our group is evaluating CD33/CD123 dual-targeted CARs to prevent antigen escape-caused relapse and may evaluate them as promising myeloablative tools for HSCT in a follow-up study.

## Conclusion

In the past few years, the progress of CAR-engineered T cells has rapidly developed and made great achievements. Nevertheless, there still exist certain limitations in this field that should not be ignored. One of the most concerning issues is that there is no convincing evidence of an AML-specific cell surface antigen that can be safely used to maximize the usefulness of CAR T cells. Admirably, many research groups are still confident and have developed numerous strategies to improve the current status of CAR T cells as a therapeutic in the AML field, such as gene-editing technology, antibodies, and combination therapies, most of which have been presented in this review. If these strategies could be successfully employed in clinical trials, the ability of CAR-expressing T cells in treating AML would be immeasurable. In addition, we hope that this review provides useful information regarding the overall progress of CAR T cell therapy in the AML and injects new ideas into future research. In conclusion, the adoptive transfer of CAR-engineered T cells represents a valuable and attractive therapeutic strategy that has the potential to provide new prospects for cancer immunotherapy.

## Abbreviations

ADCC: Antibody-dependent cell-mediated cytotoxicity
ALL: Acute lymphoblastic leukemia
allo-HSCT: Hematopoietic stem cell transplantation
AML: Acute myeloid leukemia
ATRA: All-trans retinoic acid
CAR: Chimeric antigen receptor
CDC: Complement dependent cytotoxicity
CIK: Cytokine induced killer
CRS: Cytokine release syndrome
EBV-CTL: Human Epstein Barr Virus-cytotoxic lymphocyte
EGFR: Epidermal growth factor receptor
FLT-3: Fms-like tyrosine kinase 3
FRβ: Folate receptor β
GVHD: Graft-versus-host disease
IL12: Interleukin-12
iMC: Inducible MyD88/CD40
LAG3: Lymphocyte activating 3
LSC: Leukemia stem cell
mAb: Monoclonal antibody
MHC: Major histocompatibility complex
mRNA: Messenger ribonucleic acid
NKG2D: Natural killer group 2D
PD1: Programmed death 1
scFv: Single chain variable fragment
SIRPa: Signal regulatory protein-a
TAAs: Tumor-associated antigens (TAAs)
TCR: T cell receptor
TIM-3: T-cell immunoglobulin mucin-3
TME: Tumor microenvironment (TME)
